# Metabolomics analysis in rat hearts with ischemia/reperfusion injury after diazoxide postconditioning

**DOI:** 10.3389/fmolb.2023.1196894

**Published:** 2023-05-25

**Authors:** Cen Xiang, Shoujia Yu, Qiyang Ren, Boyi Jiang, Jing Li, Donghang Zhang, Yiyong Wei

**Affiliations:** ^1^ Department of Anesthesiology, Affiliated Hospital of Zunyi Medical University, Zunyi, China; ^2^ Department of Anesthesiology, Zhuhai Hospital of Integrated Traditional Chinese and Western Medicine, Zhuhai, China; ^3^ Department of Anesthesiology, West China Hospital of Sichuan University, Chengdu, China; ^4^ Department of Anesthesiology, Longgang District Matemity and Child Healthcare Hospital of Shenzhen City (Longgang Matemity and Child Institute of Shantou University Medical College), Shenzhen, China

**Keywords:** diazoxide, metabolomics, ischaemia/reperfusion injury, myocardium, postconditioning

## Abstract

**Background:** Diazoxide is a selective mitochondrial-sensitive potassium channel opening agent that has a definite effect on reducing myocardial ischemia/reperfusion injury (MIRI). However, the exact effects of diazoxide postconditioning on the myocardial metabolome remain unclear, which might contribute to the cardioprotective effects of diazoxide postconditioning.

**Methods:** Rat hearts subjected to Langendorff perfusion were randomly assigned to the normal (Nor) group, ischemia/reperfusion (I/R) group, diazoxide (DZ) group and 5-hydroxydecanoic acid + diazoxide (5-HD + DZ) group. The heart rate (HR), left ventricular developed pressure (LVDP), left ventricular end-diastolic pressure (LVEDP), and maximum left ventricular pressure (+dp/dtmax) were recorded. The mitochondrial Flameng scores were analysed according to the ultrastructure of the ventricular myocardial tissue in the electron microscopy images. Rat hearts of each group were used to investigate the possible metabolic changes relevant to MIRI and diazoxide postconditioning.

**Results:** The cardiac function indices in the Nor group were better than those in the other groups at the end point of reperfusion, and the HR, LVDP and +dp/dt_max_ of the Nor group at T2 were significantly higher than those of the other groups. Diazoxide postconditioning significantly improved cardiac function after ischaemic injury, and the HR, LVDP and +dp/dt_max_ of the DZ group at T2 were significantly higher than those of the I/R group, which could be abolished by 5-HD. The HR, LVDP and +dp/dt_max_ of the 5-HD + DZ group at T2 were significantly lower than those of the DZ group. The myocardial tissue in the Nor group was mostly intact, while it exhibited considerable damage in the I/R group. The ultrastructural integrity of the myocardium in the DZ group was higher than that in the I/R and 5-HD + DZ groups. The mitochondrial Flameng score in the Nor group was lower than that in the I/R, DZ and 5-HD + DZ groups. The mitochondrial Flameng score in the DZ group was lower than that in the I/R and 5-HD + DZ groups. Five metabolites, namely, L-glutamic acid, L-threonine, citric acid, succinate, and nicotinic acid, were suggested to be associated with the protective effects of diazoxide postconditioning on MIRI.

**Conclusion:** Diazoxide postconditioning may improve MIRI via certain metabolic changes. This study provides resource data for future studies on metabolism relevant to diazoxide postconditioning and MIRI.

## Introduction

Myocardial ischemia/reperfusion injury (MIRI) refers to a series of complex pathophysiological changes in the myocardium after ischemia, hypoxia and blood perfusion, which can result in severe and even irreversible myocardial damage (Russo et al., 2017). MIRI is a serious threat to human health. Mitochondrial ATP-sensitive potassium channels (mitoKATP) are located in the mitochondrial intima and are considered end-effectors for alleviating MIRI (Mironova et al., 2019). Diazoxide is a specific opener of the mitoKATP channel. Studies have found that diazoxide can attenuate MIRI (Onukwufor et al., 2016; Jin et al., 2020; Chen et al., 2021). However, the exact modulation mechanism for alleviating MIRI has not been clearly illustrated.

Energy metabolism dysfunction is one of the main pathological changes during MIRI (Kolwicz et al., 2013). Hypoxia inhibits the oxidative metabolism of fatty acids, glucose, and amino acids and activates anaerobic glycolysis (Zuurbier et al., 2020). These metabolic changes largely determine the actual damage that occurs to the heart following ischemia/reperfusion. Pan et al. (2020) applied proteomic techniques to find that diazoxide postconditioning could upregulate three proteins, namely, NADH dehydrogenase (ubiquinone) flavoprotein 1 (NDUFA1), NADH-ubiquinone oxidoreductase 75 kDa subunit (NDUFS1) and 2-oxoglutarate dehydrogenase (OGDH), which are involved in maintaining mitochondrial respiratory chain function and regulating the tricarboxylic acid (TCA) cycle to maintain normal energy supply. Furthermore, mitoKATP channel opening regulates the expression of some genes (*Mt-nd6, Idh2,* and *Acadl*) related to energy metabolism and regulates the TCA cycle and fatty acid metabolism to promote ATP production (Cao et al., 2015). Therefore, mitoKATP channel opening may be involved in regulating cardiac metabolism and maintaining the balance of energy demand and supply during MIRI. However, the metabolic changes related to MIRI and diazoxide postconditioning were not well determined.

In the present study, we hypothesized that diazoxide postconditioning can impact principal metabolic pathways in hearts with MIRI. This study may provide new insights into the protective mechanism of diazoxide postconditioning in heart MIRI.

## Materials and methods

Animals. Twenty-four male Sprague-Dawley (SD) rats (250–300 g, 16–20 weeks old) were housed in a specific pathogen-free (SPF) animal facility with 12-h light/dark cycles and *ad libitum* access to food and water. All experimental procedures were approved by the Animal Care and Use Committee of Zunyi Medical University (no.KLLY (A)-2019-043) and were performed according to the Guide for the Care and Use of Laboratory Animals (National Research Council (US) Committee for the Update of the Guide for the Care and Use of Laboratory Animals, 2011).

**FIGURE 1:**
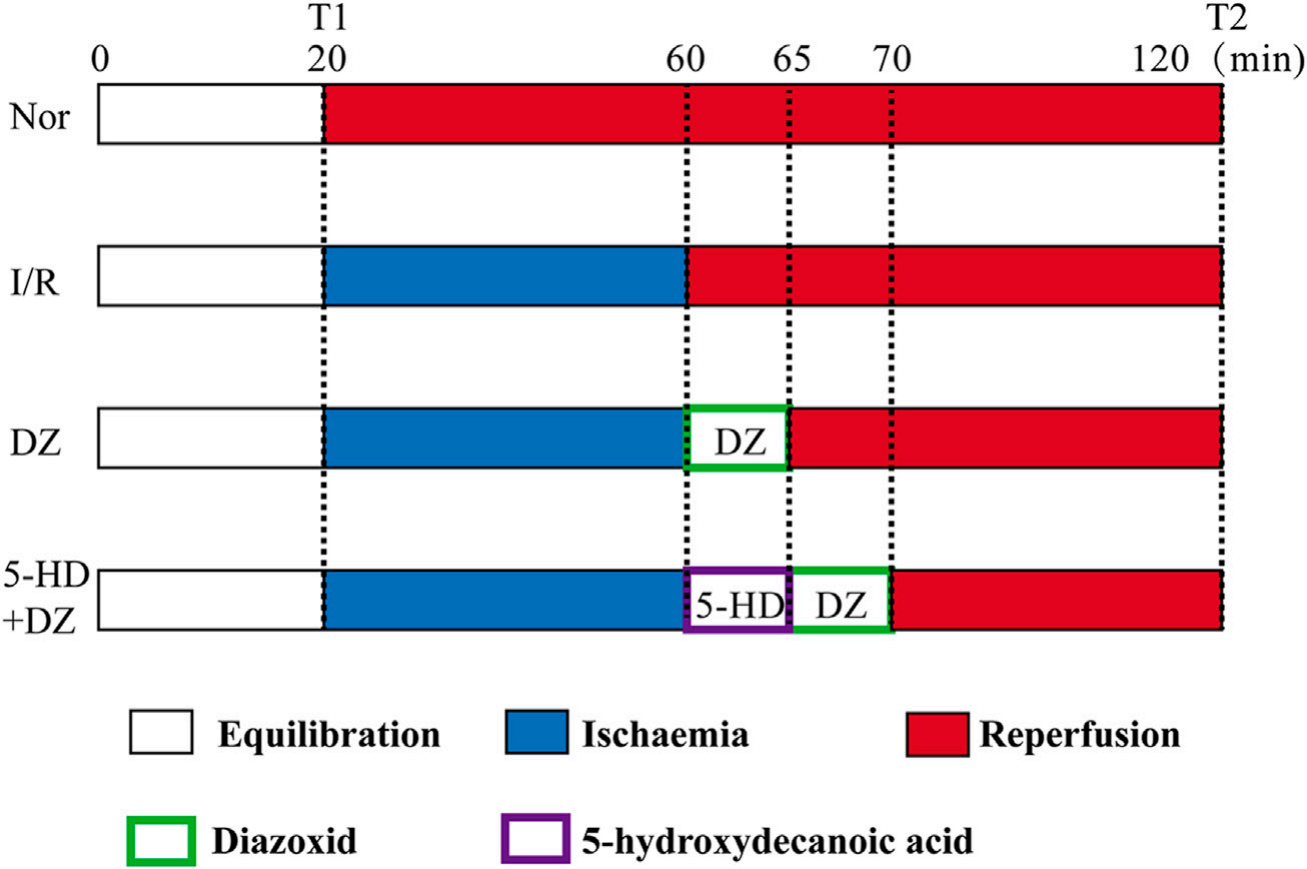
Perfusion protocol for isolated hearts.

Perfusion protocol. Rats were anaesthetized with sodium pentobarbital (40 mg/kg) by intraperitoneal injection. The heart was quickly isolated and placed in K-H solution (1.19 mM KH_2_PO_4_, 11.1 mM glucose, 4.75 mM KCl, 1.19 mM MgCl_2_•6H_2_O, 2.50 mM CaCl_2_ and 24.80 mM NaHCO_3_, 118.00 mM NaCl, 4°C, pH 7.40). Then, the heart was rapidly connected to a Langendorff perfusion system. All hearts were perfused by a Langendorff apparatus with K-H solution for 20 min for equilibration. The rat hearts were randomly divided into four groups, including the Nor, I/R, DZ and 5-HD + DZ groups (n = 6/group). Hearts in the Nor group were continuously perfused with K-H solution with oxygen for 120 min. After 20 min of equilibration, hearts in the I/R w group ere perfused with St. Thomas solution (1.20 mM CaCl_2_, 110.00 mM NaCl, 16.00 mM MgCl_2_, 10.0 mM NaHCO_3_, 16.00 mM KCl, 4°C) for ischemia for 40 min and then were perfused with K-H solution for 60 min. After ischemia, hearts in the DZ group were perfused with K-H solution containing diazoxide (50 μM) for 5 min before reperfusion and then with K-H solution for 55 min. Hearts in the 5-HD + DZ group were perfused with 5-hydroxydecanote (50 μM in K-H solution) for 5 min, perfused with diazoxide (50 μM) for 5 min, and then with K-H solution for 50 min. The protocols are outlined in Figure 1. At the end of reperfusion, the ventricular tissues were collected and stored at −80°C. At the end of equilibration (T1) and reperfusion (T2), the heart rate (HR), left ventricular developed pressure (LVDP), left ventricular end-diastolic pressure (LVEDP), and maximum left ventricular pressure (+dp/dt_max_) were recorded by the Langendorff perfusion system.

Transmission electron microscopy (TEM)*.* Myocardial tissue (1 mm^3^) at the end of reperfusion was fixed with 2.5% glutaraldehyde electron microscopic fixative, rinsed with PBS and then fixed with 1% osmic acid. The tissues were dehydrated with acetone and embedded using epoxy resin (35°C overnight, 45°C for 8 h, 60°C for 48 h). Myocardial sections were stained with uranyl acetate and lead citrate for 20 min. The myocardial ultrastructure was examined using an electron microscope (HITACHI-H7500, Hitachi, Japan), and mitochondrial damage was evaluated using the Flameng scoring method for each group (n = 6) (Flameng et al., 1980).

Metabonomics analysis. In total, 25 mg of myocardial tissue samples and 800 μl of methanol and acetonitrile were mixed for each group (n = 6). The mixture was sonicated for 10 min and centrifuged at 25,000 rpm for 15 min. The resulting supernatants were transferred to LC-MS vials and stored at −80°C. Quality control (QC) samples were prepared by mixing equal aliquots of the supernatants from all of the samples. LC-MS/MS analysis was performed using an UHPLC system (Waters 2D; Waters, United States) with a high-resolution mass spectrometer (Q Exactive; Thermo Fisher Scientific, United States). Mobile phase A was 0.1% formic acid in water for the positive ion mode and 10 mM ammonia formate in water for the negative ion mode, and mobile phase B was 10 mM ammonia formate in 95% methanol.

Raw MS data were filtered using the following criterion: less than 50% of all sample numbers in a group contained a metabolite (QC samples were also taken as a group). Missing values were replaced by half of the minimum value in the dataset by default. OSI-SMMS (Version 1.0, Dalian Chem Data Solution Information Technology Co. Ltd.) was used for the self-built database after XCMS (Version 3.2) data processing. The repeated metabolites from the positive and negative ion modes were merged. The variable importance in projection (VIP) score of the PLS-DA model was applied to rank the metabolites that best distinguished between comparisons. A t-test was also used for univariate analysis to screen differential metabolites. Metabolites with *p* < 0.05 and VIP ≥1 were considered differential metabolites between comparisons. Metabolites were mapped to KEGG metabolic pathways for pathway and enrichment analyses. *p* < 0.05 was considered statistically significant. Pathways meeting this condition were defined as significantly enriched pathways for differential metabolites.

Statistical analysis. Statistical analyses were performed using SPSS (Version 22.0, IBM Corp., Armonk, NY, and United States). Normally distributed data are presented as the mean ± SD. Groups were compared using Student’s t test (normally distributed data). Two-way ANOVA followed by Sidak’s multiple comparisons test was used to compare haemodynamic parameters at the same time point in different groups. Comparisons of the Flameng scores among different groups were conducted using the Kruskal-Wallis test followed by Dunn’s *post hoc* test for multiple comparisons. To identify correlations between metabolite levels and the LVDP, stepwise multivariate linear regression was used, and Pearson’s correlation analysis was applied using MetaboAnalyst 4.0.

## Result

**FIGURE 2:**
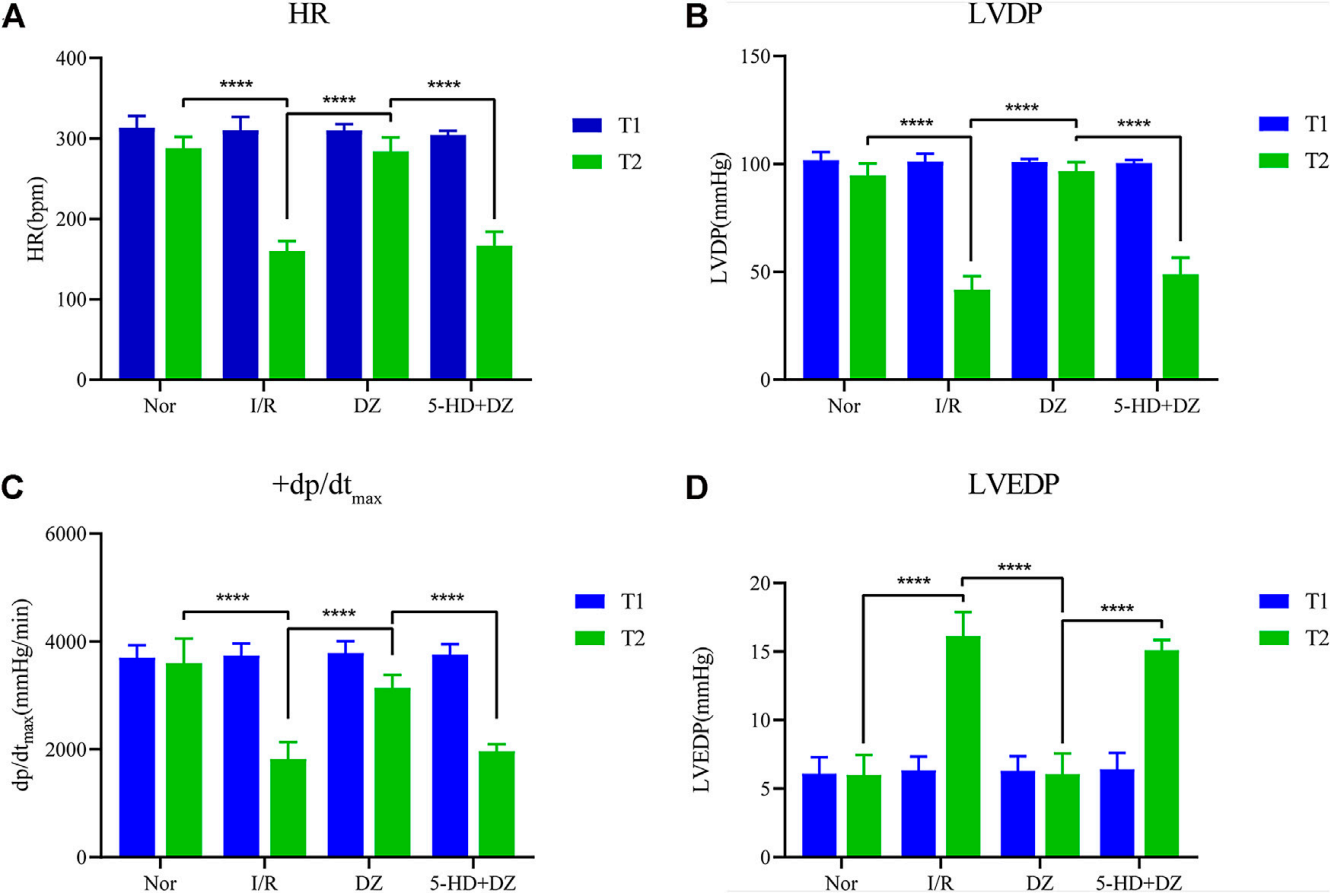
Haemodynamic parameters. Diazoxide postconditioning improves HR **(A)**, LVDP **(B)**, dp/dtmax **(C)** and LVEDP **(D)** after MIRI, whereas 5-HD eliminates the effect (comparisons at different time points in the same group and comparisons at the same time point in different groups were conducted using two-way ANOVA followed by Sidak’s *post hoc* test). ***p < 0.001. HR, heart rate; dp/dtmax, maximum rate of the rise in intraventricular pressure; LVDP, left ventricular developed pressure; LVEDP, left ventricular end-diastolic pressure.

Diazoxide postconditioning improves cardiac function. There was no statistical significance in the comparison of HR, LVDP, +dp/dt_max_ and LVEDP among the four groups at T1. The HR, LVDP and +dp/dt_max_ of the Nor group and the DZ group at T2 were significantly higher than those of the I/R and 5-HD + DZ groups (Figures 2A–C), whereas the LVEDP of the Nor group and the DZ group at T2 was lower than that of the I/R and 5-HD + DZ groups (Figure 2D). These findings suggested that diazoxide postconditioning improved I/R-induced haemodynamic dysfunction, which was abolished by treatment with its blocker 5-HD.

**FIGURE 3:**
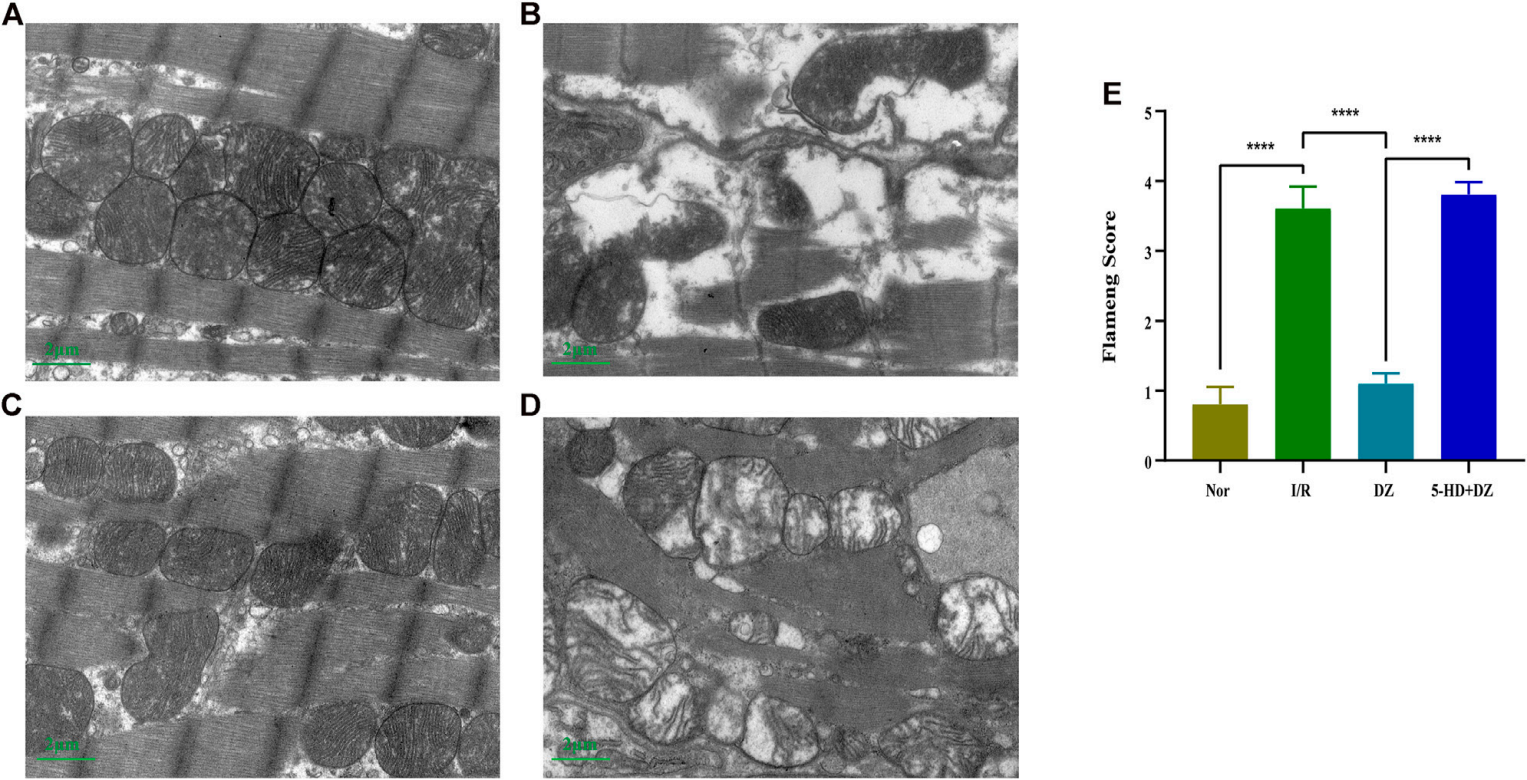
Myocardial ultrastructure and the Flameng score. At the end of reperfusion, the ultrastructure of cardiomyocytes was analysed by transmission electron microscopy (magnification: ×10,000) in the Nor **(A)**, I/R **(B)**, DZ **(C)** and 5-HD + DZ **(D)** groups. Flameng scores of mitochondria **(E)**. Comparisons among the different groups were conducted using the Kruskal-Wallis test followed by Dunn’s *post hoc* test. ***p < 0.001. I/R, ischemia/reperfusion group; DZ, diazoxide postconditioning group; 5-HD + DZ, 5-HD (mitoK_ATP_ blocker) + diazoxide group.

Myocardial ultrastructure and the Flameng score. The myofilaments in the I/R and 5-HD + DZ groups were ruptured, dissolved and disordered. Moreover, the mitochondria were dissolved and destroyed (Figures 3B, D). In the DZ group, myocardial morphology was basically normal, and fewer myofilaments were dissolved. Most mitochondria in the DZ group were intact, but a few of them were slightly swollen (Figures 3A, C). The Flameng score in the DZ group was lower than that in the I/R group (*p* < 0.001) (Figure 3E). However, the Flameng score in the 5-HD + DZ group was significantly higher than that in the DZ group (*p* < 0.001) (Figure 3E).

**FIGURE 4:**
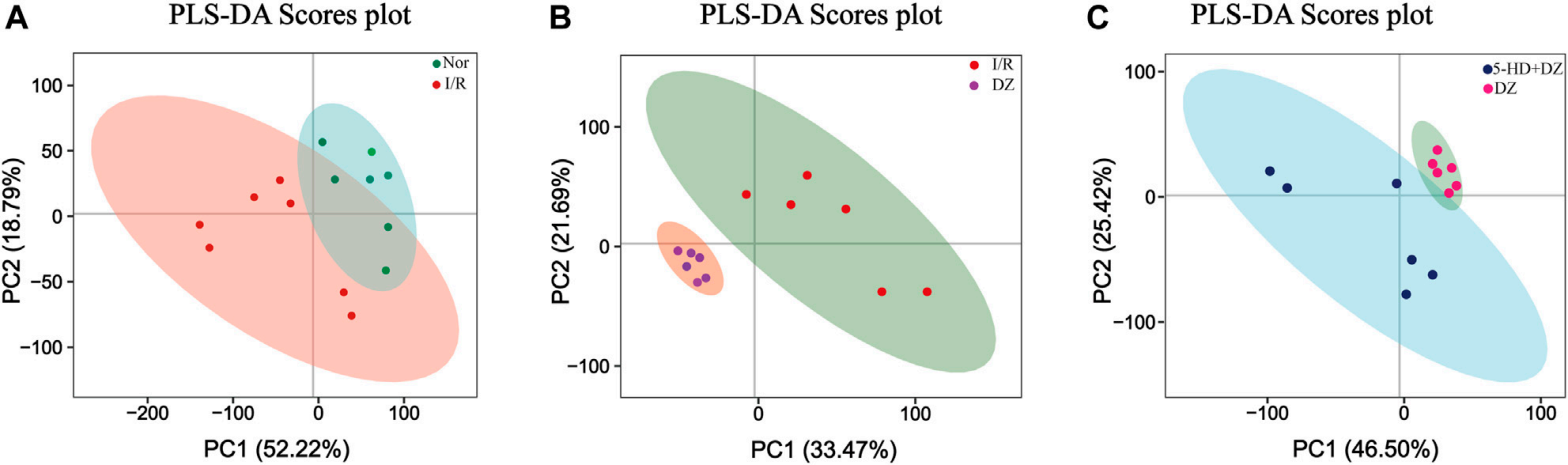
Score plot of PLS-DA. Score plot in the Nor group and I/R group **(A)** score plot in the DZ group and I/R group **(B)** score plot in the DZ group and 5-HD + DZ group **(C)**. PC1, the first principal component score. PC2, the second principal component score. PLS-DA, partial least squares discriminant analysis.

Multivariate statistical analysis. A partial least squares discriminant (PLS-DA) model was built using a dataset including the four groups of samples. R2Y and Q2 represent the interpretation rate of the Y matrix and the predicted variation, respectively, with parameters as follows: R2Y = 0.90, Q2 = 0.26 (Nor group *vs*. I/R group); R2Y = 0.98, Q2 = 0.84 (DZ group *vs*. I/R group); and R2Y = 0.95, Q2 = 0.63 (DZ group *vs*. 5-HD + DZ group). The score plots revealed that each class was well separated, suggesting that the PLS-DA model successfully discriminated samples according to their underlying metabolic profiles (Figures 4A–C).

**FIGURE 5:**
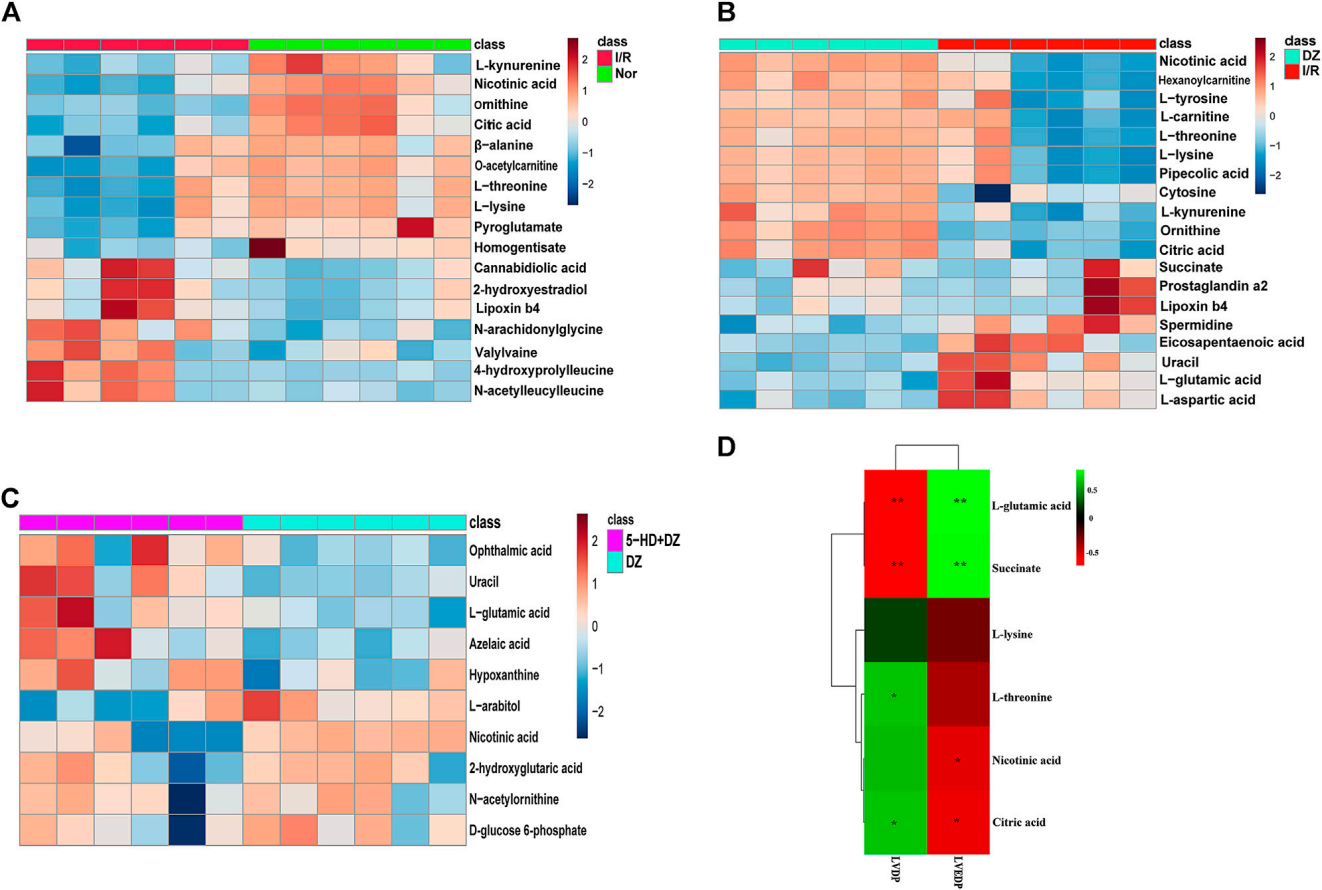
Heatmap of differential metabolites and correlations between metabolite levels and LVDP. Heatmap of differential metabolites in the Nor group *vs*. I/R group **(A)**, DZ group *vs*. I/R group **(B)**, and DZ group *vs*. 5-HD + DZ group **(C)**. Heatmap showing the correlations of metabolites to LVDP and LVEDP **(E)**. *p < 0.05, **p < 0.001.

Identification of potential regulated metabolites. The PLS-DA model was used to screen differential metabolites. Metabolites with *p* < 0.05 and VIP ≥1 were considered differential metabolites between comparisons. The levels of ten metabolites were significantly decreased after myocardial ischemia/reperfusion injury, while the levels of eight metabolites increased (Table 1). The levels of eight metabolites were significantly decreased after diazoxide postconditioning, while ten metabolites increased (Table 2). The levels of five metabolites were significantly decreased after 5-hydroxydecanoic acid and diazoxide postconditioning, while six metabolites increased (Table 3). The significantly changed metabolites between the Nor and I/R groups are presented as heatmaps in the Supplementary Material (Figure 5A). The significantly changed metabolites between the DZ and I/R groups are presented as heatmaps in the Supplementary Material (Figure 5B). The significantly changed metabolites between the DZ and 5-HD + DZ groups are presented as heatmaps in the Supplementary Material (Figure 5C). Our results showed that the levels of nicotinic acid, citric acid, L-threonine and L-lysine in the Nor and DZ groups were higher than those in the I/R group. The levels of nicotinic acid in the DZ group were higher than those in the I/R and 5-HD + DZ groups. The levels of L-glutamic acid in the DZ group were lower than those in the I/R and 5-HD + DZ groups.

**TABLE 1:** Significantly differential metabolites in heart tissues between Nor and I/R group.

Metabolites	Formula	VIP	*p*-value	FC
4-hydroxyprolylleucine	C_11_ H_20_ N_2_ O_4_	1.5048	0.0384	58.6213
N-acetylleucylleucine	C_14_ H_26_ N_2_ O_4_	1.423	0.0424	47.5131
2-hydroxyestradiol	C_18_ H_24_ O_3_	1.422	0.0443	29.5835
N-arachidonylglycine	C_22_ H_35_ N O_3_	1.2318	0.0046	3.5885
Cannabidiolic acid	C_22_ H_30_ O_4_	1.2007	0.0119	6.0837
Lipoxin b4	C_20_ H_32_ O_5_	1.1962	0.0404	9.6321
Prostaglandin g2	C_20_ H_32_ O_6_	1.1885	0.0406	17.930
Valylvaline	C_10_ H_20_ N_2_ O_3_	1.0222	0.0384	3.3415
Nicotinic acid	C_6_ H_5_ N O_2_	2.1749	0.0005	−0.0449
O-acetylcarnitine	C_9_ H_17_ N O_4_	2.0184	0.0150	−0.1905
Ornithine	C_5_ H_12_ N_2_ O_2_	1.9882	0.0021	−0.0992
Pyroglutamate	C_5_ H_7_ N O_3_	1.7696	0.0091	−0.1066
Citric acid	C_6_ H_8_ O_7_	1.6698	0.0008	−0.1503
Homogentisate	C_8_ H_8_ O_4_	1.544	0.0247	−0.1291
L-kynurenine	C_10_ H_12_ N_2_ O_3_	1.4219	0.0116	−0.1606
L-threonine	C_4_ H_9_ N O_3_	1.1585	0.0237	−0.4020
L-lysine	C_6_ H_14_ N_2_ O_2_	1.112	0.0203	−0.4253
Β-alanine	C_3_ H_7_ N O_2_	1.1018	0.0448	−0.3569

FC, Fold change; VIP, Variable importance.

**TABLE 2:** Significantly differential metabolites in heart tissues between DZ *vs*. I/R group.

Metabolites	Formula	VIP	*p*-value	FC
Uracil	C_4_ H_4_ N_2_ O_2_	1.7776	0.0034	0.0809
Eicosapentaenoic acid	C_20_ H_30_ O_2_	1.4318	0.0163	0.2139
L-aspartic acid	C_4_ H_7_ N O_4_	1.3743	0.0053	0.1461
Prostaglandin a2	C_20_ H_30_ O_4_	1.2555	0.048	0.1325
Lipoxin b4	C_20_ H_32_ O_5_	1.2416	0.0485	0.0995
L-glutamic acid	C_5_ H_9_ N O_4_	1.2415	0.0117	0.2673
Succinate	C_4_ H_6_ O_4_	1.224	0.0139	0.2668
Spermidine	C_7_ H_19_ N_3_	1.1164	0.0044	0.4652
Nicotinic acid	C_6_ H_5_ N O_2_	2.3879	0.0011	−14.7018
Hexanoylcarnitine	C_13_ H_25_ N O_4_	2.1384	0.0095	−4.9302
Ornithine	C_5_ H_12_ N_2_ O_2_	2.0173	0.0000	−8.6706
L-carnitine	C_7_ H_15_ N O_3_	2.0143	0.0465	−1.8073
Citric acid	C_6_ H_8_ O_7_	1.8272	0.0001	−5.7788
L-threonine	C_4_ H_9_ N O_3_	1.3456	0.0387	−2.0369
L-lysine	C_6_ H_14_ N_2_ O_2_	1.3272	0.0309	−2.0230
Pipecolic acid	C_6_ H_11_ N O_2_	1.3338	0.0307	−2.0331
Cytosine	C_4_ H_5_ N_3_ O	1.3068	0.0199	−2.9574
L-kynurenine	C_10_ H_12_ N_2_ O_3_	1.2496	0.0004	−3.3334
L-tyrosine	C_9_ H_11_ N O_3_	1.1905	0.0397	−1.8692

FC, Fold change; VIP, Variable importance.

**TABLE 3:** Significantly differential metabolites in heart tissues between DZ *vs*. 5-HD + DZ group.

Metabolites	Formula	VIP	P	FC
Uracil	C_4_ H_4_ N_2_ O_2_	1.6671	0.0221	7.4379
Sebacic acid	C_10_ H_18_ O_4_	1.5291	0.0127	2.9046
Ophthalmic acid	C_11_ H_19_ N_3_ O_6_	1.2602	0.039	2.2042
Azelaic acid	C_9_ H_16_ O_4_	1.2346	0.0283	1.9060
L-glutamic acid	C_5_ H_9_ N O_4_	1.183	0.0313	2.7131
Hypoxanthine	C_5_ H_4_ N_4_ O	1.0309	0.0462	1.5543
Nicotinic acid	C_6_ H_5_ N O_2_	3.0196	0.0316	−0.2919
2-hydroxyglutaric acid	C_5_ H_8_ O_5_	1.8662	0.0142	−0.3227
N-acetylornithine	C_7_ H_14_ N_2_ O_3_	1.154	0.0389	−0.5583
L-arabitol	C_5_ H_12_ O_5_	1.1321	0.0378	−0.5208
D-glucose 6-phosphate	C_6_ H_13_ O_9_ P	1.0887	0.0313	−0.5526

FC, Fold change; VIP, Variable importanc.

**FIGURE 6:**
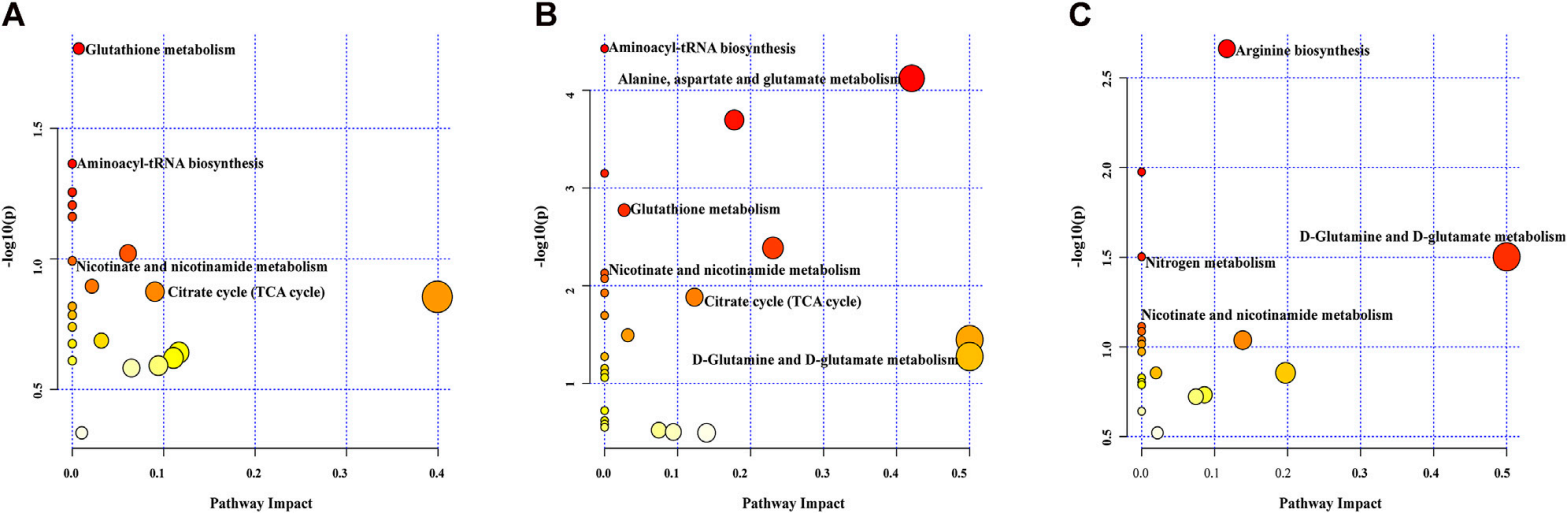
Pathway analysis of different metabolites. Nor group *vs*. I/R group **(A)**, DZ group *vs*. I/R group **(B)**, DZ group *vs*. 5-HD + DZ group **(C)**.

Biological pathway analysis. Pathway analysis was applied to investigate the biological functions of the altered metabolites. Multiple metabolic pathways were impacted after MIRI and diazoxide postconditioning in both the Nor group and the I/R group (Figure 6A), both the DZ group and the I/R group (Figure 6B), and both the DZ group and the 5-HD + DZ group (Figure 6C), mainly including the tricarboxylic acid cycle, nicotinate and nicotinamide metabolism, glutamate metabolism, glutathione metabolism, and aminoacyl-tRNA biosynthesis.

Changes in five metabolites are relevant to diazoxide postconditioning. Correlation analysis was used to determine the metabolites related to diazoxide postconditioning in MIRI. Finally, we identified five metabolites that may be relevant to diazoxide postconditioning: L-glutamic acid (LVDP: *p* = 0.0036), L-threonine (LVDP: *p* = 0.0400), citric acid (LVDP: *p* = 0.0400), succinate (LVDP: *p* = 0.0036), and nicotinic acid (LVDP: *p* = 0.0317) (Figure 5D).

## Discussion

In this study, diazoxide postconditioning was found to significantly reduce myocardial ischemia-reperfusion injury, which is consistent with our previous results (Cao et al., 2015; Pan et al., 2020; Chen et al., 2021). The present study indicated that MIRI disturbed multiple metabolic changes, including decreased levels of citrate, nicotinate, L-threonine, and L-lysine. However, diazoxide postconditioning increased the levels of these metabolites. In addition, MIRI also increased the levels of succinate and L-glutamic acid. However, the levels of these metabolites were decreased after diazoxide postconditioning. Our results suggest that the TCA cycle and amino acid metabolism might be relevant to MIRI and the myocardial protective mechanism of diazoxide postconditioning.

The TCA cycle, also known as the citric acid cycle (CAC) or Krebs cycle, is a significant pathway for various nutrients, including glucose, fatty acids, and amnio acids, and eventually produces ATP through oxidative phosphorylation (LaNoue et al., 1970). Citric acid is an important intermediate in the TCA cycle and is produced from oxaloacetate and acetyl-CoA.

In the TCA cycle, citric acid is converted into α-ketoglutarate (α-KG) via isocitrate by isocitrate dehydrogenase (IDH) (Akram, 2014). Citric acid plays a significant role in inflammatory response-related pathways and is connected with several important proinflammatory mediators, including NO, ROS and prostaglandin E_2_ (PGE_2_) (Williams and O’Neill, 2018). A previous study found that the level of citric acid was decreased after MIRI (Mu et al., 2017), which was consistent with our findings.

In the present research, the citric acid content was increased after diazoxide postconditioning. In the TCA cycle, succinate is produced from α-KG via succinyl CoA synthase and converted into fumarate via succinate dehydrogenase (SDH) (Huang and Millar, 2013). During ischemia, the accumulation of succinate is caused by reversal of SDH, which might convert fumarate to succinate. Following reperfusion, abundant succinate is rapidly oxidized and drives reverse electron transport (RET) to generate massive reactive oxygen species (ROS) at mitochondrial complex I (Chouchani et al., 2014; Kula-Alwar et al., 2019). Early studies revealed that extensive ROS induce serious acute damage and variations in pathology (Granger and Kvietys, 2015; Bystrom et al., 2017; Cadenas, 2018). ROS trigger mitochondrial permeability transition pore (mPTP) opening in conjunction with alterations in Ca^2+^ levels and physiological pH, aggravating MIRI (Cadenas, 2018; Bugger and Pfeil, 2020). Evidence indicates that inhibiting SDH reduces the content of succinate and ROS production, thus protecting cardiac function from MIRI (Valls-Lacalle et al., 2018; Xu et al., 2018). Our previous result showed that diazoxide postconditioning can decrease ROS level (Chen et al., 2021). This study showed that the level of succinate decreased after diazoxide postconditioning, which may be involved in inhibiting SDH (Anastacio et al., 2013). The cardioprotective effect of diazoxide postconditioning may be associated with regulating CAC intermediates and maintaining the normal function of the CAC.

Nicotinic acid (NA) and nicotinamide, which are known as vitamin B3, are precursors of nicotinamide adenine dinucleotide (NAD^+^) and nicotinamide adenine phosphate (NADP) (Cantó et al., 2015). NAD^+^ plays a significant role in diverse metabolic pathways, including the CAC and oxidative phosphorylation (OXPHOS), glycolysis, fermentation, cell signalling and inflammatory pathways (Yang and Sauve, 2016). NAD^+^ is converted into NADH by accepting hydride groups in normal oxidation. Then, NADH is oxidized to produce ATP in mitochondria through OXPHOS, which is responsible for 95% of the ATP production in the heart (Matasic et al., 2018). The function of OXPHOS was impaired by MIRI due to a series of enzymes, such as NADH oxidase or mitochondrial ROS release. Many studies have confirmed that increasing NAD^+^ during MIRI could mitigate oxidative stress and alleviate myocardial injury (Trueblood et al., 2000; Yamamoto et al., 2014; Tai et al., 2015). In the present study, the NA content significantly decreased after I/R, while the NA content increased after diazoxide postconditioning, indicating that diazoxide may exert protective effects by regulating NA and NAD^+^ concentrations.

Glutamate is an important excitatory neurotransmitter in the central nervous system and is associated with cerebral I/R injury (Kim et al., 2017; Leung et al., 2020; Song et al., 2020). In addition, glutamate receptors and transporters exist in peripheral tissues, such as the heart, lung, kidney and liver (Du et al., 2016). Currently, the effect of glutamate on the heart is still controversial. Studies have claimed that exogenous glutamate supplementation may alleviate injury caused by myocardial ischemia (Kristiansen et al., 2008; Sufit et al., 2012 no date). However, several studies hold the contrary opinion. One study demonstrated that the concentration of glutamate was elevated after 15 min of reperfusion in a rat cardiac transplantation model (Venturini et al., 2009). Additionally, Sun et al. (2014) claimed that the serum glutamate concentration increased after I/R and mediated ventricular arrhythmias by inducing Ca^2+^ accumulation, which may be mediated by initiating voltage-dependent Ca^2+^ channels and NMDA receptor channels (Piccirillo et al., 2020). Yokoyama et al. (2019) showed that mitoKATP opening can suppress glutamate release to reduce neuronal injury induced by I/R in the brain. Consistently, our results demonstrate that the level of L-glutamic acid in the DZ group was lower than that in the I/R and 5-HD + DZ groups. The mechanism of glutamate release in MIRI is quite complicated and should be determined in future studies.

L-Threonine is an essential amino acid in the human body and plays a significant role in lipid metabolism (Chen et al., 2022). Under anaerobic conditions, L-threonine is converted to keto acid, which produces ATP via substrate-level phosphorylation to provide a source of energy to the cells (Simanshu et al., 2007). Research has reported that L-threonine may participate in the innate immune response by activating the NF-κB signalling pathway and suppressing sirtuin-1 (SIRT1) (Wu et al., 2018). Recently, studies have proven that both the NF-κB signalling pathway and SIRT1 are associated with MIRI (Xu et al., 2021; Aghamohammad et al., 2022; Xiao et al., 2022). The cardiovascular protective effect of the NF-κB signalling pathway is related to regulating the inflammatory response (Dong et al., 2022). SIRT1, an NAD^+^-dependent deacetylase, plays a significant role in antioxidative stress activity (Zhang et al., 2017). Lysine is an indispensable amino acid. Zhang et al. (2019) reported that L-lysine supplementation may ameliorate proinflammatory changes by reducing lipid peroxidation and proinflammatory mediators, including tumour necrosis factor α (TNFα), interleukin-8 and macrophage inhibitory factor, to protect against acute lung injury. Furthermore, lysine is one of the amino acid groups most susceptible to modification (Moreno-Yruela et al., 2022). In recent years, lysine methylation, lysine succinylation, and lysine acetylation have been closely associated with cardiovascular disease and have provided a new theoretical basis for the treatment of heart disease (Boylston et al., 2015; Yi et al., 2017; Herr et al., 2020). Although the effects of L-threonine and L-lysine on MIRI have not been reported, our study found that the level of L-threonine after MIRI was decreased, which can be alleviated by diazoxide postconditioning, suggesting that these metabolite changes might be relevant to MIRI and the myocardial protective mechanism of diazoxide postconditioning.

### Study limitations

The present study has several limitations. First, we should add 5-HD group to make our results more rigor and we will consider this in future study. Seconed, the solated hearts of langendorff may not be an appropriate model for studying myocardial ischemia/reperfusion injury. It will be interesting to explore the effects of diazoxide postconditioning on ischemia/reperfusion injury using a classic ischemia/reperfusion method, and further compared the results between different ischemia/reperfusion methods. Third, we did not perform *in vivo* experiments to confirm the metabolic changes and protective effects of diazoxide postconditioning in MIRI. Four, we did not validate the function of these identified metabolites or the associated pathways in MIRI.

## Conclusion

In summary, the present study indicated that diazoxide postconditioning may improve MIRI via certain metabolic changes, including changes in the levels of citrate, nicotinic acid, L-glutamic acid, L-threonine and L-lysine. This study provides resource data for future studies on metabolism relevant to diazoxide postconditioning and MIRI.

## Data Availability

The original contributions presented in the study are included in the article/Supplementary Materials, further inquiries can be directed to the corresponding author.
